# Characterization of the complete mitochondrial genome of *Cottiusculus nihonkaiensis* (Scorpaeniformes, Cottidae) and phylogenetic studies of Scorpaeniformes

**DOI:** 10.1080/23802359.2020.1866456

**Published:** 2021-02-08

**Authors:** Lele Yang, Kehua Zhu, Jiaqi Fang, Liqin Liu, Zhenming Lü

**Affiliations:** a National Engineering Laboratory of Marine Germplasm Resources Exploration and Utilization, Zhejiang Ocean University, Zhoushan, China; b National Engineering Research Center for Facilitated Marine Aquaculture, Marine Science and Technology College, Zhejiang Ocean University, Zhoushan, China

**Keywords:** *Cottiusculus nihonkaiensis*, mitochondrial genome, phylogenetic analysis, Scorpaeniformes

## Abstract

In this study, the complete mitochondrial genome of *Cottiusculus nihonkaiensis* was presented, and we also discussed its mitochondrial characteristics. The full length of the mitochondrial genome was 16,612 bp, containing 13 protein-coding genes (PCGs), two ribosomal RNAs (12S and 16S), 22 transfer RNA genes (tRNA), one non-coding control region (CR) and one origin of replication on the light-strand. Overall base composition of the complete mitochondrial DNA was 26.4% A, 17.4% G, 31.5% C, 24.7% T. The phylogenetic tree suggested that *C. nihonkaiensis* shared the most recent common ancestor with *Gymnocanthus herzensteini, Gymnocanthus intermedius* and *Gymnocanthus tricuspis*.

*Cottiusculus nihonkaiensis* in the family Cottidae (Scorpaeniformes) is distributed the Sea of Japan, Sea of Okhotsk and Pacific coast of Hokkaido (Kai and Nakabo 2009). It is a temperate marine fish, inhabiting in the bottom sea area (Kai and Yamanaka 2019). In this study, we described the complete mitochondrial genome of *C. nihonkaiensis* and analyzed the phylogenetic relationship of Scorpaeniformes, to gain its molecular information and thus contribute to facilitate future studies on population genetic structure and phylogenetic relationships.

The sample of *C. nihonkaiensis* was collected from the East China Sea (25°54′15′N, 117°05′24′E) and stored in 95% alcohol, then kept in the laboratory of Zhejiang Ocean University with accession number 20170927SJL25. Total genomic DNA was extracted using a phenol-chloroform extraction protocol (Kchl et al. 2005). Subsequently, based on the existing complete mitochondrial gene of *Gymnocanthus herzensteini* (KX148474), 18 pairs of primers were designed, the samples were amplified by PCR, and then sequenced using Sanger sequencing technology. NOVOPlasty software was used to assemble the mitogenomes, the mistake parameter was set by default (Dierckxsens et al. 2017). The boundaries of ribosomal RNA genes were identified by a BLAST search (http://blast.ncbi.nlm.nih.gov). The locations of the transfer RNAs and protein-coding genes were identified using the program tRNAscan-SE version 2.0 (http://trna.ucsc.edu/tRNAscan-SE/) (Li et al. 2013) and MITOS WebServer (http://mitos2.bioinf.uni-leipzig.de/index.py), respectively. The one origin of replication on the light-strand and control region were determined by the proposed secondary structures and sequence homology. The whole mitochondrial genome of *C. nihonkaiensis* was a closed double-stranded circular molecule consisting of 16,612 bp (GenBank accession number: MK224511), which was very similar to other typical vertebrate mitochondria (Miya et al. 2001; Zhu et al. 2018a, 2018b). The complete mitochondrial genome contains 13 protein-coding genes (PCGs), 22 transfer RNAs (tRNA) genes, two ribosomal RNA genes (12S rRNA and 16S rRNA), a putative control region (CR) and one origin of replication on the light-strand (OL). The overall base composition was 26.4% A, 17.4% G, 31.5% C, 24.7% T, respectively, with a slight AT bias (51.1%). *C. nihonkaiensis* mitochondrial genes were mostly encoded on the heavy strand, and only ND6 and eight tRNA (Gln, Ala, Asn, Cys, Tyr, Ser, Glu and Pro) genes on the light strand coding. The start codons of the 13 PCGs encoding genes were ATG except for COI which was GTG, which is quite common in vertebrate mtDNA (He et al. 2015). Most of the stop codons were TAA or T––, the stop codon of ND2 was CTA and the gene with TTA as the stop codon was COIII. Most of the tRNAs could form a common cloverleaf secondary structure, except tRNA^Ser (GCT)^ gene without DHU stem (Han et al. 2016). The lengths of the two rRNA genes were 947 bp (12S rRNA) and 1,692 bp (16S rRNA), respectively, which located between the tRNA^Phe^ and tRNA^Leu (TAA)^ and separated by the tRNA^Val^ gene. The length of the control region was 858 bp, located between tRNA^phe^ and tRNA^Pro^.

In order to obtain the position and kinship of the *C. nihonkaiensis* within Scorpaeniformes, we constructed phylogenetic trees of Scorpaeniformes based on maximum likelihood (ML) method (Figure 1). According to the Akaike Information Criteria (AIC), the most suitable nucleotide sequence model was selected through MrModeltest 2.3 (Bozdogan 1987), and finally the most suitable model was GTR + I + G. The ML phylogenetic tree based on 13 PCGs of 36 species using the software RAxML 8.0 (Alexandros 2014). The results showed that *C. nihonkaiensis* shared the most recent common ancestor with *Gymnocanthus herzensteini*, *Gymnocanthus intermedius* and *Gymnocanthus tricuspis*.

**Figure 1.**
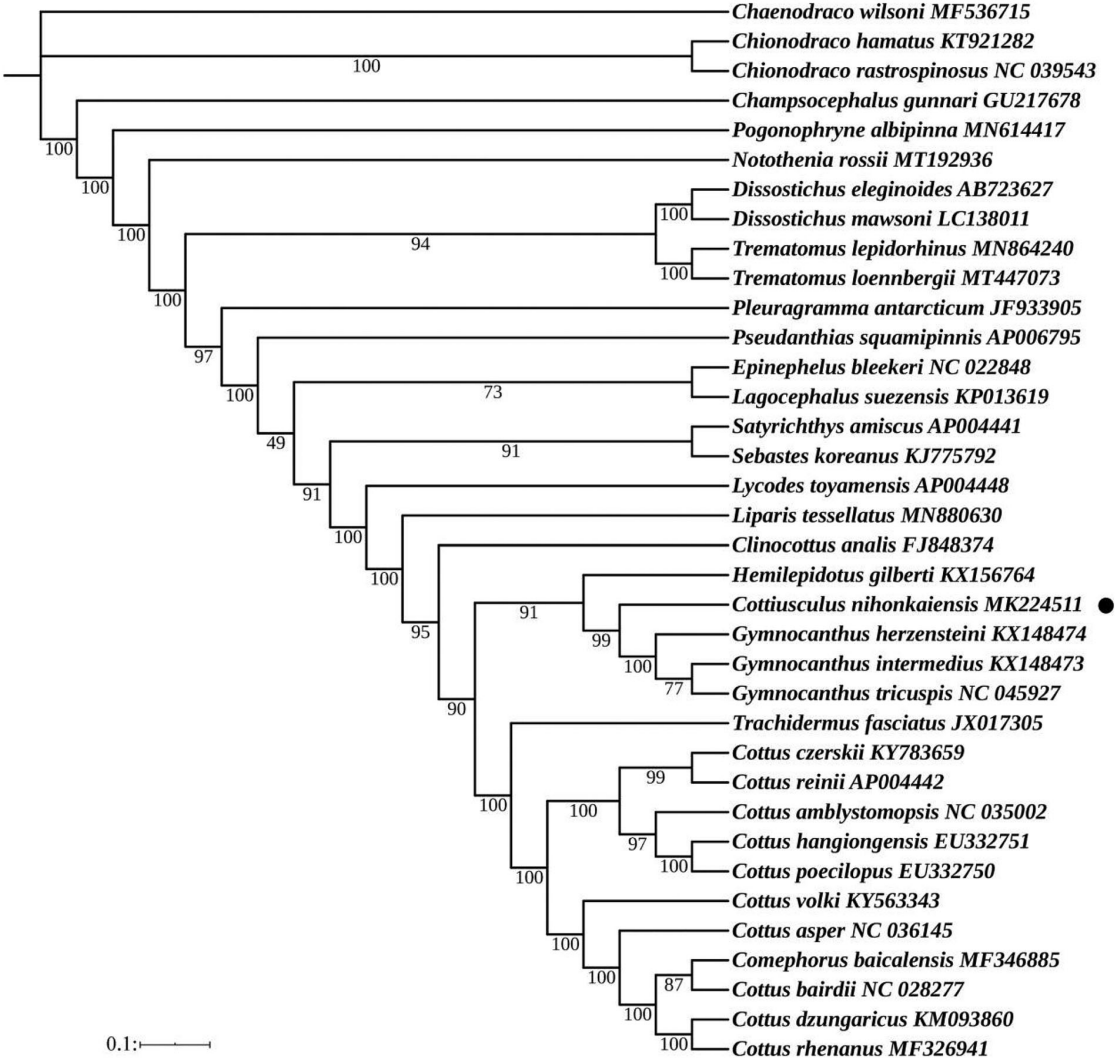
Phylogenetic analysis based on the nucleotide sequences of the 13 PCGs in the mitogenome. Maximum Likelihood analyses (bootstrap support with 1000 replications) are shown next to nodes. The number after the species name was the GenBank accession number. The genome sequence in this study was labeled with a black dot.

## Data Availability

The data that support the findings of this study are openly available in “NCBI” athttps://www.ncbi.nlm.nih.gov/nuccore/MK224511. GenBank accession number: MK224511.1.
